# Synchronization of Spontaneous Active Motility of Hair Cell Bundles

**DOI:** 10.1371/journal.pone.0141764

**Published:** 2015-11-05

**Authors:** Tracy-Ying Zhang, Seung Ji, Dolores Bozovic

**Affiliations:** 1 Department of Physics and Astronomy, University of California Los Angeles, Los Angeles, California, United States of America; 2 California NanoSystems Institute, University of California Los Angeles, Los Angeles, California, United States of America; 3 Department of Physical Science, Los Angeles Mission College, Sylmar, California, United States of America; University of British Columbia, CANADA

## Abstract

Hair cells of the inner ear exhibit an active process, believed to be crucial for achieving the sensitivity of auditory and vestibular detection. One of the manifestations of the active process is the occurrence of spontaneous hair bundle oscillations *in vitro*. Hair bundles are coupled by overlying membranes *in vivo*; hence, explaining the potential role of innate bundle motility in the generation of otoacoustic emissions requires an understanding of the effects of coupling on the active bundle dynamics. We used microbeads to connect small groups of hair cell bundles, using *in vitro* preparations that maintain their innate oscillations. Our experiments demonstrate robust synchronization of spontaneous oscillations, with either 1:1 or multi-mode phase-locking. The frequency of synchronized oscillation was found to be near the mean of the innate frequencies of individual bundles. Coupling also led to an improved regularity of entrained oscillations, demonstrated by an increase in the quality factor.

## Introduction

Hair cells of the inner ear are the mechanical sensors that detect air- and ground-borne vibrations and transduce them into electrical signals (reviewed in [1–4]). A hair cell consists of a cell soma and an array of columnar structures called the stereovilli, which comprise the hair bundle. The actin-filled stereovilli are arranged in rows of increasing height and are coupled together by tip links [5]. The tips of the hair bundles are connected to an overlying membrane, termed the otolithic membrane in the bullfrog sacculus. An incoming stimulus induces a shearing motion between the overlying membrane and the tissue in which the cells are embedded, deflecting the bundle and thus increasing the tension on the tip links. Mechanically sensitive ion channels that are physically coupled to the tip links open in response and allow the inflow of cations [6, 7].

When stimulated by an incoming signal, the hair bundle oscillates in a viscous fluid environment. An internal active process, which pumps energy to amplify the sound-induced vibrations, has been suggested to overcome viscous dissipation [8]. Two different mechanisms have been proposed for the amplification process, including somatic electromotility [9–13] and active hair bundle motility [4, 14–18]. The active process has been experimentally demonstrated *in vitro* (reviewed in [19, 20]). Under conditions that mimic the natural environment, individual hair bundles can exhibit innate oscillations [21, 22] in the absence of any input. This spontaneous motility has been extensively studied in hair cell bundles of the anuran sacculus and can be explained by two processes [2, 3]. Opening and closing of the mechanically sensitive ion channels lead to bistability in the position of the bundle. In addition, an adaptation mechanism mediated by myosin motors provides a feedback mechanism [5, 23–27], poising the bundle in the regime of highest sensitivity. Interplay between these two processes leads to a relaxation oscillation. Comparisons to the fluctuation-dissipation theory have proven that the observed spontaneous motility requires an underlying energy-consuming process [28].

While the role of an amplifier *in vivo* is still under debate [29], a number of observations indicate its presence [19, 20]. One of the signatures of an active process, observed in many species, is the phenomenon of spontaneous otoacoustic emission (SOAE) [30–32]. These are weak sounds emitted by the ear in the absence of an applied stimulus. *In vivo*, most hair cell bundles are connected to overlying gelatinous structures, with varying strength and extent of the coupling observed in different species [32]. Theoretical studies explored the effects of connecting a number of nonlinear oscillators and proposed that under certain conditions, active bundle motility should synchronize [33]. Entrained motility of bundles, or small groups of bundles, was shown to lead to frequency clustering. Numerical simulations based on this frequency clustering [34] reproduced the measured peaks in the emission spectra [31, 35]. However, the connection between the spontaneous oscillations *in vitro* and the presence of SOAEs *in vivo* has not been proven experimentally.

Synchronization of active motility was predicted for systems where the individual oscillators exhibit identical or similar frequencies. One study introduced coupling between an oscillatory hair bundle and its cyber clone, a numerical simulation replicating the oscillation profile of the biological bundle [36]. Their results showed that the hair cell and its cyber clone synchronized their active motility. A theoretical study showed that larger systems of inter-connected hair bundles [33] exhibit enhanced sensitivity and frequency tuning of the response.

Different phenomena could be expected in a system that shows a broad dispersion of frequencies among the individual cells. With sufficiently large discrepancies in frequencies of the constituent oscillators, coupling could lead to amplitude death and render the system quiescent [37]. The theoretical model predicting amplitude death is consistent with the observed quiescence of saccular hair bundles, fully coupled by the overlying otolithic membrane [38].

We explore whether active innate motility will synchronize in small groups of coupled hair bundles. A hybrid preparation combines live and spontaneously oscillating hair bundles with an artificial coupling element. The amphibian sacculus, an organ specializing in detection of low-frequency auditory and vestibular signals [39], provides the biological epithelium for this study. The frequencies of spontaneous oscillation in this end organ show no spatial dependence but are uniformly and randomly distributed across the epithelium [40]. Therefore, neighboring hair cells can exhibit quite disparate characteristic frequencies. We hence study synchronization of spontaneous oscillation on systems exhibiting different dispersions of characteristic frequencies. We compare our experimental results to a numerical model of coupled nonlinear oscillators.

## Materials and Methods

### Biological preparation

Protocols for animal care and euthanasia were approved by the University of California Los Angeles Chancellor’s Animal Research Committee (protocol number ARC 2006-043-13C), in accordance with federal and state regulations. Prior to dissection procedures, animals were euthanized while under pentobarbital anesthesia. The sacculi were excised from the inner ears of the American bullfrog (*Rana catesbeiana*). The preparation was mounted in a two-compartment chamber, simulating the fluid separation of the *in vivo* environment, with artificial perilymph solution (in mM: 110 Na^+^, 2 K^+^, 1.5 Ca^2+^, 113 Cl^-^, 3 d-glucose, 1 Na^+^ pyruvate, 1 creatine, and 5 HEPES) on the basal side and artificial endolymph (2 Na^+^, 118 K^+^, 0.25 Ca^2+^, 118 Cl^-^, 3 d-glucose, and 5 HEPES) on the apical side. For both solutions, the pH and osmolality were adjusted to be 7.3 and 230 mmol/kg, respectively. Solutions were oxygenated immediately prior to use. The overlying otolithic membrane was digested by exposing the apical surface of the epithelium to 50μg/mL Collagenase (Sigma Aldrich) for 8 minutes and gently removed.

### Artificial coupling of hair bundles

Mechanical coupling of hair bundles was provided with 50 mm diameter polystyrene microspheres (Corpuscular Inc.). The spheres were coated with concanavalin A (Sigma Aldrich), a highly charged polymer which enhances adhesion to stereovilli. After incubation in concanavalin A, the polystyrene particles were centrifuged and re-suspended in artificial endolymph, at a concentration of 3.5mg/ml. The beads were introduced into the top compartment of the recording chamber and allowed to settle onto the saccular preparation. Post deposition, fluid in the top compartment was replaced with artificial endolymph. After motion of the coupled bundles was recorded, the beads were suctioned off with a pipette.

Bundles were found to oscillate at comparable amplitudes under coupled and uncoupled conditions. Since spontaneous oscillation was shown to correlate closely with opening and closing of the transduction channels [41], the presence of the microsphere did not significantly interfere with the transduction process.

### Imaging hair bundle and bead motion

Hair bundles were imaged in a top-down configuration with an upright light microscope (Olympus B51W). The image was further magnified to ~500x and projected onto a Complementary Metal Oxide Semiconductor (CMOS) camera (Photron SA 1.1). Recordings were acquired at 250 to 1000 frames per second. Motion was tracked with software written in MatLab (Mathworks), which performs a center-of-mass calculation on the intensity profiles of the bundles. For each hair bundle, this calculation was averaged over at least 15 rows of pixels to extract its mean position in each frame of the recording. The position vs. time traces were smoothed by a moving average to remove higher-frequency (>150 Hz) noise.

Given the size of the beads, images were obtained at two focal planes—the equatorial plane of the sphere and the plane spanning the tips of the stereovilli. The polystyrene material was found to be sufficiently transparent to allow imaging of hair bundles through it. Also visible within the focal plane of the bundles were occasional dark spots within the bead that result from non-uniformity of the polystyrene. These spots were imaged to allow the tracking of bead motion in the same focal plane as the cell bundles. To improve the precision of the tracking, recordings of multiple dark spots within the field of view were averaged. Additionally, by tracking three spots spread over the focal plane, and measuring the distortions in the triangle defined by the points over time, we could estimate the rotation of the bead to be <4°; the bead motion was mostly confined to the x-y plane.

### Data analysis

An automated routine [42] was used to detect positive and negative transitions in the position of the hair bundles and/or overlying bead, in each low-pass filtered motion trace. It was shown [21] that these transitions correspond to the opening and closing of the transduction channels, respectively. The period between two consecutive positive transitions defines the instantaneous period of one cycle, from which we obtain the instantaneous frequency. To quantify the regularity of the oscillation frequency in each trace, the probability density of the instantaneous frequency was obtained using kernel density estimation (Matlab function ksdensity). We compute the quality factor of the distribution: Q = F_peak_/FWHM, where F_peak_ is the frequency of the peak in the density function, and FWHM is the full width at half maximum. An example of this procedure is shown in the supplemental information (S1 Fig).

With the focal plane at the level of the stereovilli, our CMOS recordings provided 8–10 hair bundles in the field of view, 3–5 of which were underneath a microsphere. We obtained simultaneous motion traces for each of the bundles, as well as the dark spots within the bead. To quantify the degree of synchronization between various pairs of bundles, we computed the cross correlation function between the traces. The cross correlation function *f*(t) between x(τ) and y(τ) is defined as $f\left(t\right)=\frac{<x\left(\tau \right),y\left(\tau +t\right)>}{\surd <x\left(\tau \right),x\left(\tau \right)><y\left(\tau \right),y\left(\tau \right)>}$, where <> is the inner product of vectors. The correlation coefficient is taken to be the peak value of *f*(t), and is normalized so that the correlation coefficient of a function with itself is 1. Recordings of bead motion, obtained in the same focal plane, were used to calculate the correlation between the bead and each of the bundles underneath. The distribution of correlation coefficients, compiled from 8 preparations, shows a cluster of correlated bundles (panel A in S2 Fig). Based on the distribution, we selected 0.5 to be the threshold correlation coefficient, with higher values indicating synchronization. Phase lags of hair bundle motion with respect to that of the microsphere were within the time resolution of the recordings, for all synchronized hair cells.

The duration of the recordings varied from 1 to 11 seconds in length. To verify that the degree of synchronization did not vary significantly over time, we calculated the correlation coefficient in moving time windows, each 0.5 second long, for recordings longer than 5 seconds. Fluctuations in the correlation coefficient were below 0.1, for synchronized cells, and remained above 0.5 throughout the records. An example of the variation of correlation with time is shown in the supplement (panel B in S2 Fig).

To determine the instantaneous phase of oscillation, we obtained the complex form z(t) of the oscillation y(t) by letting *z* = −*Hilbert*(*y*) + *iy*, where Hilbert(y) is the Hilbert transform of y. The instantaneous phase θ is given by $\theta =\text{arctan}\left(\frac{Imag\left(z\right)}{Real\left(z\right)}\right)$.

### Coupling strength

To estimate the coupling strength between the hair bundles and the bead, we used a glass fiber to impose lateral displacements on the bead, and recorded its response, as well as that of the bundles. Spontaneous bundle oscillations were suppressed by introducing artificial perilymph on the apical side of the preparation. Sequential bursts of sine waves were sent, each at a different frequency, and the amplitude and phase of the response were measured for each stimulus segment. The measured responses yielded values for the elastic and viscous coupling coefficients, K and ξ, between the individual bundles and the bead (detailed in S1 File). Peaks in the histogram distributions of the measured values provided estimates of the coupling coefficients; the measurement was repeated for four groups of hair bundles, yielding the average results K = 2.5 +/-1.1 mN/m and ξ = 2.8+/-0.7 μN*s/m.

## Results

### Synchronization of active motility by artificial coupling

Prior work has shown that frequencies of spontaneous oscillation are randomly distributed throughout the saccular epithelium [40]. Consistent with this finding, the preparations studied displayed a significant dispersion in the innate frequencies of the cells. Synchronization was observed even with very disparate frequencies of the individual oscillators.

**Fig 1:**
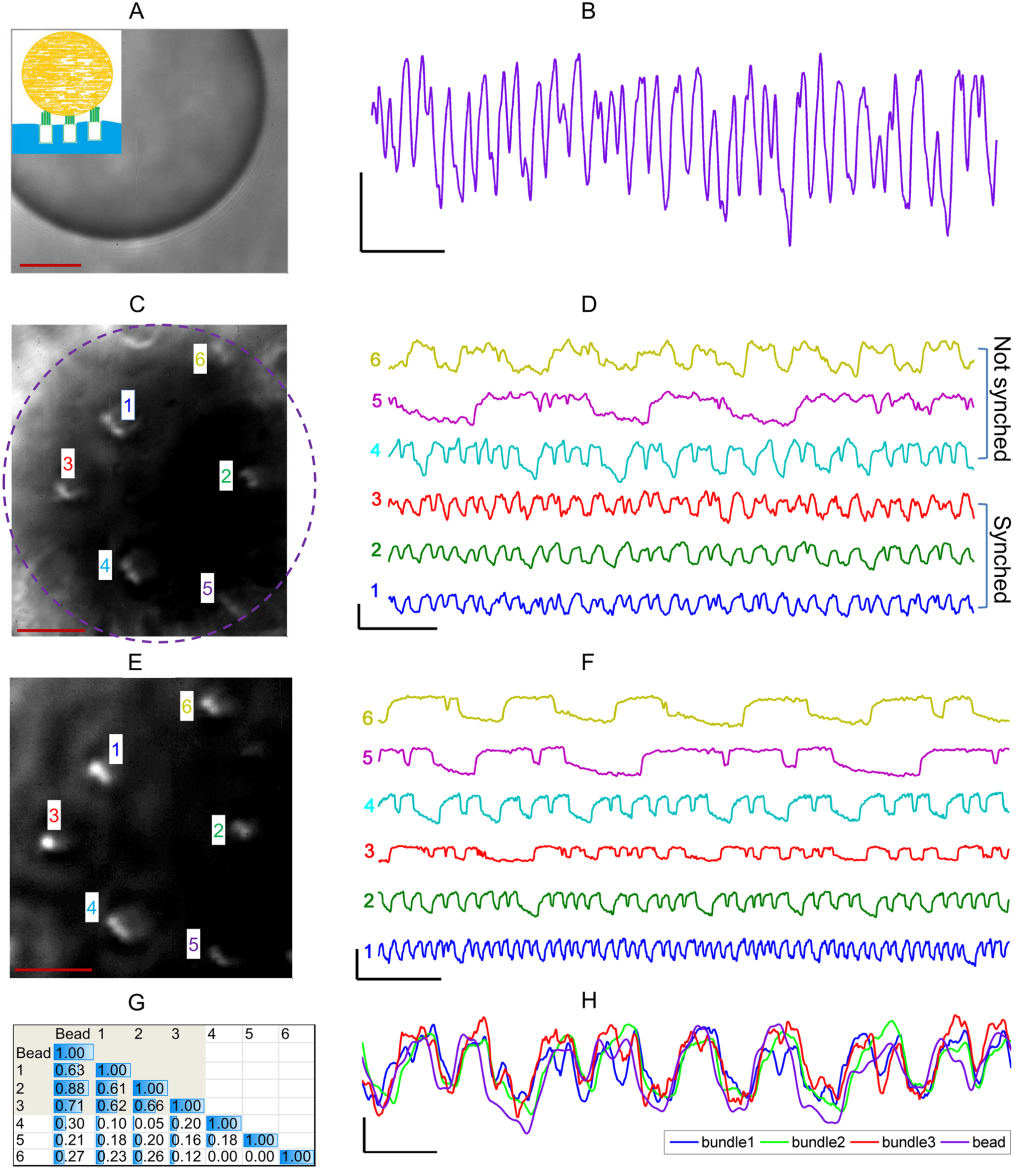
Synchronization of spontaneous oscillations. **(A)** Image of a polystyrene sphere at the equatorial plane of focus, obtained in a top-down view. Scale bar = 10 μm. Inset shows a schematic of the sphere on top of the bundles, in a sideways view. **(B)** Motion trace of the microsphere obtained at the equatorial plane. Scale bar x = 400 ms, y = 40 nm. **(C)** Top-down view of hair bundles, imaged through the overlying polystyrene sphere. Dashed line marks the projection of the rim of the sphere onto this focal plane. Scale bar = 10 μm. **(D)** Traces of spontaneous motion of the hair bundles shown in **C**, denoted by the corresponding numbers. Bundles 1–3 synchronized their motion to each other, whereas those near the rim of the bead (bundles 4–6) did not. Scale bar x = 400 ms, y = 50 nm. **(E)** Top-down view of the hair bundles, obtained after removal of the bead. Scale bar = 10 μm. **(F)** Traces of spontaneous bundle motility, recorded after bead removal. Scale bar x = 400 ms, y = 50 nm. **(G)** Table of the normalized correlation coefficients for the bundle oscillations and those of the bead. Bundles 1–3 were synchronized, and 4–6 were not. **(H)** Overlaid traces of hair bundle (1–3) and bead motility, demonstrating phase-locked oscillation. Scale bar x = 100 ms, y = 20 nm.

Fig 1 shows an example illustrating synchronization of active hair bundle motility by the overlying bead. The power generated by the collective phase-locked motion of the bundles was found to be sufficient to drive the relatively large overlying microsphere. One set of recordings was obtained with the plane of focus at the equatorial level of the bead (Fig 1A), which revealed a robust spontaneous oscillation (Fig 1B).

To determine the degree to which individual bundles were synchronized by the coupling, we imaged the bundles through the overlying bead, with the plane of focus aimed at the tips of the stereovilli (Fig 1C). The position of each bundle was tracked separately to obtain simultaneous traces of motion (Fig 1D). All six bundles underneath the bead exhibited spontaneous motility. The three hair cells near the rim of the microsphere showed no entrainment to neighboring cells, indicating that they were not coupled (see S1 File), as expected from the spherical shape of the bead. Three hair bundles that were more centrally located synchronized their active oscillation.

For the last set of recordings, the bead was removed by suction through a pipette. Active motility in decoupled hair cells was recorded (see Fig 1E and 1F). In the absence of the overlying structure, no innate correlation was observed in the motility of the hair bundles, consistent with prior findings.

We characterized the degree of synchronization among coupled oscillatory hair bundles by calculating the maximum in the normalized cross-correlation function for each pair (Fig 1G). To extract the motion of the bead in the same recording as the bundles, we tracked dark spots in the image of the microsphere. Overlaid traces of hair bundle oscillations and those of the bead clearly indicate that they were mode-locked and in phase (Fig 1H). Higher correlation was typically observed between a hair bundle and the bead to which it is coupled, compared to correlations between pairs of bundles.

Synchronization was studied in seven preparations, on groups of spontaneously oscillating hair bundles with an overlying polystyrene sphere. Typically, 3–4 bundles synchronized their active motility and led to an entrained motion of the bead, yielding correlation coefficients above 0.5. Bundles near the rims of the microspheres did not synchronize their motion. Hair bundles that synchronized their oscillations were mostly located within 16 mm of the bead’s center (see S1 File) due to the spherical shape of the bead and the heights of the stereovilli.

The entrained hair bundles could clearly provide sufficient power to overcome the viscous damping and drive spontaneous oscillations of the beads, with amplitudes up to 80 nm. Phase lags of bundle motion with respect to that of the microsphere were within the time resolution of tracking.

### Oscillation frequency of the coupled system

**Fig 2:**
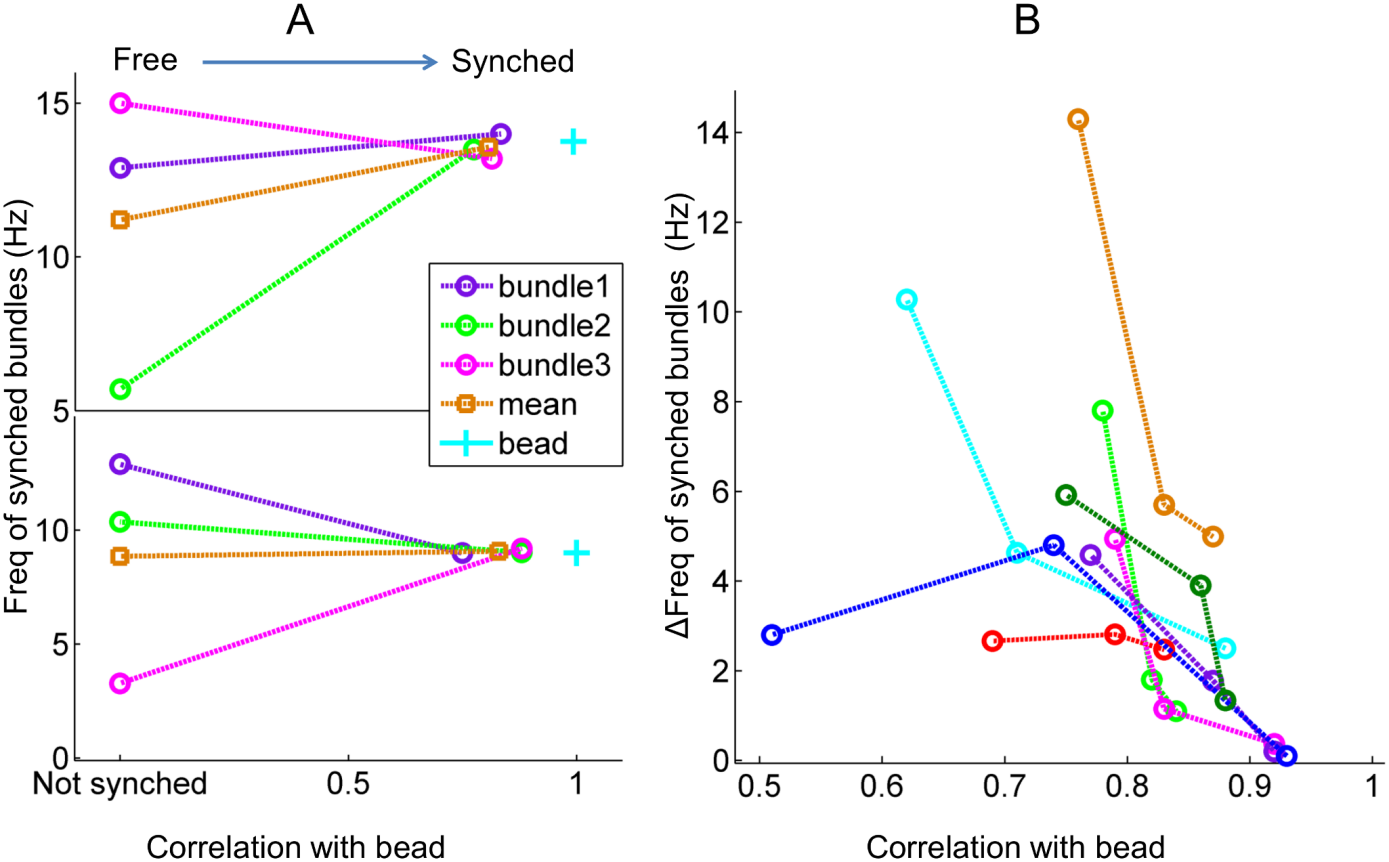
Frequency of the synchronized system. **(A)** Each panel represents a group of synchronized hair cell bundles. For each group, the frequency values of bundle oscillations, with and without an overlying bead, is plotted versus the correlation coefficient of the bundle motion with respect to that of the bead. For synchronized bundles, their frequencies of oscillation converge to the mean frequency of the group. **(B)** Change in the bundle frequency (ΔFreq) versus the correlation coefficient. ΔFreq is defined as the absolute value of the difference between a bundle’s oscillation frequency in the synchronized and unsynchronized state. Each color represents a synchronized group (8 groups total) of hair bundles, and each point represents a bundle in the group. ΔFreq shows a decreasing trend with the correlation coefficient.

Innate frequencies of the individual bundles were determined by characterizing their oscillations in the absence of an attached bead, as illustrated in Fig 1F. Comparing the innate frequencies to those of the synchronized bundles (two examples shown in Fig 2A), we observed a consistent pattern: the synchronized oscillators converged to the group’s mean frequency, which shifted very little as a result of the coupling element. The frequency of the bead oscillation, determined from a separate recording in the equatorial plane of focus, reflected the mean frequency of the individual bundles.

We next examined the dependence of the induced frequency shift on the correlation coefficient between the bundle and the bead (Fig 2B). Within each group of hair bundles, higher correlation coefficients corresponded to smaller ΔFreq, indicating that bundles synchronized more readily when the innate frequencies matched more closely the mean frequency of the group.

### Enhanced regularity of bundle oscillations in the coupled system

**Fig 3:**
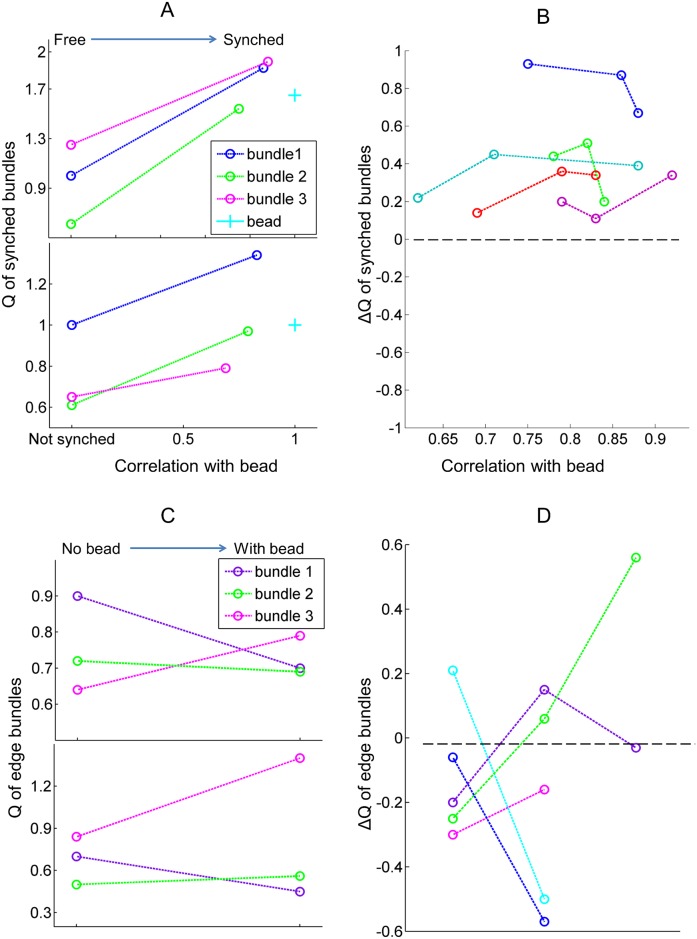
Enhanced regularity of spontaneous oscillations. **(A)** Each panel represents a synchronized group of oscillators. For each group, the quality factor of the oscillations exhibited by hair bundles, when synchronized by the bead and upon its removal, is plotted against the bundle’s coefficient of correlation with the bead. Synchronization increases the regularity of the oscillations. **(B)** ΔQ versus the correlation coefficient. ΔQ, defined as Q_synchronized_-Q_unsynchronized_, is measured for each bundle. Each color represents a synchronized group (5 groups total), and each point represents a bundle in the group. ΔQ is always positive, indicating that the synchronized system exhibits an enhanced regularity of oscillation. **(C)** Each panel shows a group of unsynchronized bundles, positioned near the rim of the bead. For each group, we compare the quality factors of the bundles with and without the bead present. The quality factor either increases or decreases, showing no consistent trend. **(D)** ΔQ, obtained for groups of unsynchronized bundles near the rims of the beads. ΔQ was either positive or negative, showing no consistent trends.

Synchronized oscillations were observed to be more regular than the noisy innate oscillations of individual hair bundles. To measure the reduction in the variation of the oscillation frequency, we calculated the quality factors of the instantaneous frequency for all spontaneously oscillating hair bundles (n = 5), with and without the presence of an overlying bead (2 examples shown in Fig 3A). Quality factors were found to be consistently higher under coupled conditions, compared to those extracted from the bundles’ innate motility. As a control, the same analysis was performed for the unsynchronized edge bundles (see, for example, bundles 4–6 in Fig 1). Fig 3C shows the quality factors of spontaneous oscillations of edge bundles, with and without the presence of an overlying microsphere. For these groups of cells, quality factors of the individual bundles showed either an increase or a decrease upon the deposition and removal of the bead, with no overall trend, indicating that the regularity of the innate oscillation was not affected.

This measurement was performed for five groups of cells with recordings longer than five seconds. All of the groups showed an improvement in the regularity of spontaneous oscillation (positive ΔQ) as a result of synchronization (Fig 3B). In comparison, the unsynchronized edge bundles showed no trend in ΔQ (Fig 3D). The synchronized system exhibited an enhanced regularity of spontaneous oscillation.

### Multi-mode phase locking

**Fig 4:**
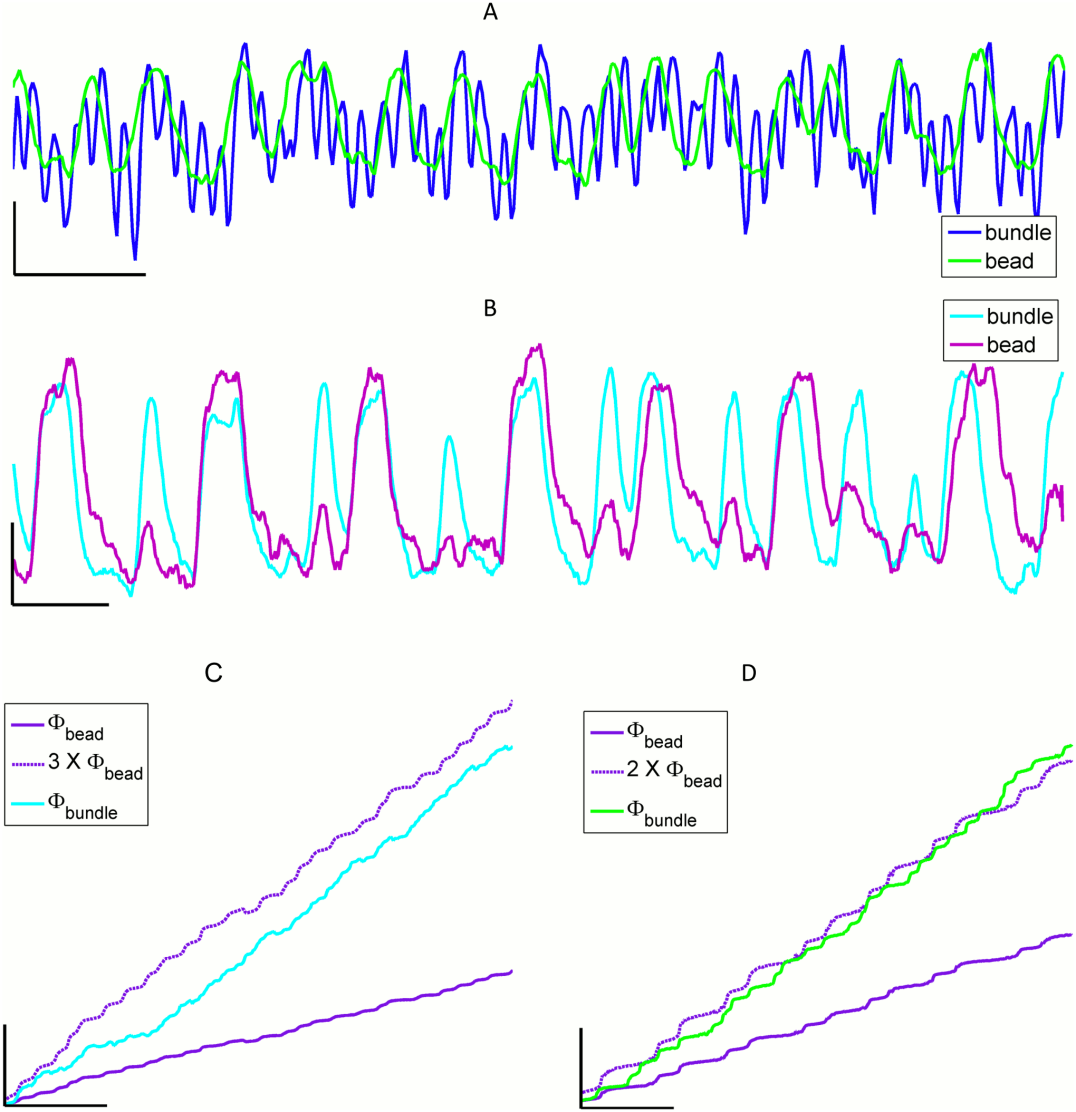
High-order mode-locking. **(A)** Traces of motion for a hair bundle and bead pair, showing 3:1 mode locking. Scale bar x = 200 ms, y = 30 nm. **(B)** Traces of motion for a bundle and bead pair, with 2:1 mode locking. Scale bar x = 100 ms, y = 30 nm. **(C)** The unwrapped phase of the pair shown in part A. Instantaneous phase of the bundle (Φ_bundle_) increases faster than that of the overlying bead (Φ_bead_). Multiplying Φ_bead_ by 3 leads to a largely parallel evolution of the phases with time. Scale bar x = 300 ms, y = 50 rad. **(D)** The unwrapped phase of the pair shown in part B. Multiplying Φ_bead_ by 2 leads to a largely parallel evolution of the two phases with time. Scale bar x = 200 ms, y = 20 rad.

Mode-locking in 1:1 ratio of frequencies, as illustrated in the traces of bundle motion shown in Fig 1, was observed in clusters of up to three hair bundles. In instances where four cells synchronized their motion, one bundle in the coupled group was found to exhibit high-order mode-locking. Fig 4 shows two examples, with overlaid traces demonstrating multi-mode phase-locking: Fig 4A shows a hair bundle whose oscillation mode-locked to that of the bead in a 3:1 ratio of frequencies, with intermittent flicker to other mode-locking ratios. Fig 4B shows an example of 2:1 mode-locking. For bundles that exhibited high-order entrainment, the correlation coefficients between their active motility and the motion of the bead were found to be lower than for 1:1 entrainment, between 0.4–0.6.

Examining the unwrapped phases of the oscillations further illustrates high-order mode-locking (Fig 4C and 4D). The instantaneous phase of the bundle increased at a higher rate than that of the bead, and the two phases diverged over time. If the bead’s phase was multiplied by an appropriate integer n, the time traces of the two phases were found to be parallel.

## Numerical Model of Coupled Hair Bundles

### System of coupled nonlinear equations

We also explore coupled non-linear oscillators theoretically. We describe the dynamics of each individual hair bundle by the normal form equation of the Andronov-Hopf bifurcation. In the complex form, the equation is given by:

$$\frac{\text{dz}}{\text{dt}}=\left(i\omega +\mu \right)z-{\left|z\right|}^{2}z$$

,where the ω is the frequency of spontaneous oscillation and the μ is the control parameter. This model undergoes the Andronov-Hopf bifurcation when μ = 0. We define the real part of the equation to be the lateral deflection of the bundle, and the imaginary part to reflect the internal dynamics of the hair cell. The parameters m and w represent the negative stiffness and the characteristic frequency of the bundle, respectively.

$$\frac{\text{dx}}{\text{dt}}=\mu \text{ x}-\omega \;\text{y}-\left({\text{x}}^{2}+{\text{y}}^{2}\right)\text{x}$$

$$\frac{\text{dy}}{\text{dt}}=\mu \;\text{x}+\omega \;\text{x}-\left({\text{x}}^{2}+{\text{y}}^{2}\right)\text{y}$$

This simple model has been shown to capture the main characteristics of hair bundle response [43, 44].

**Fig 5:**
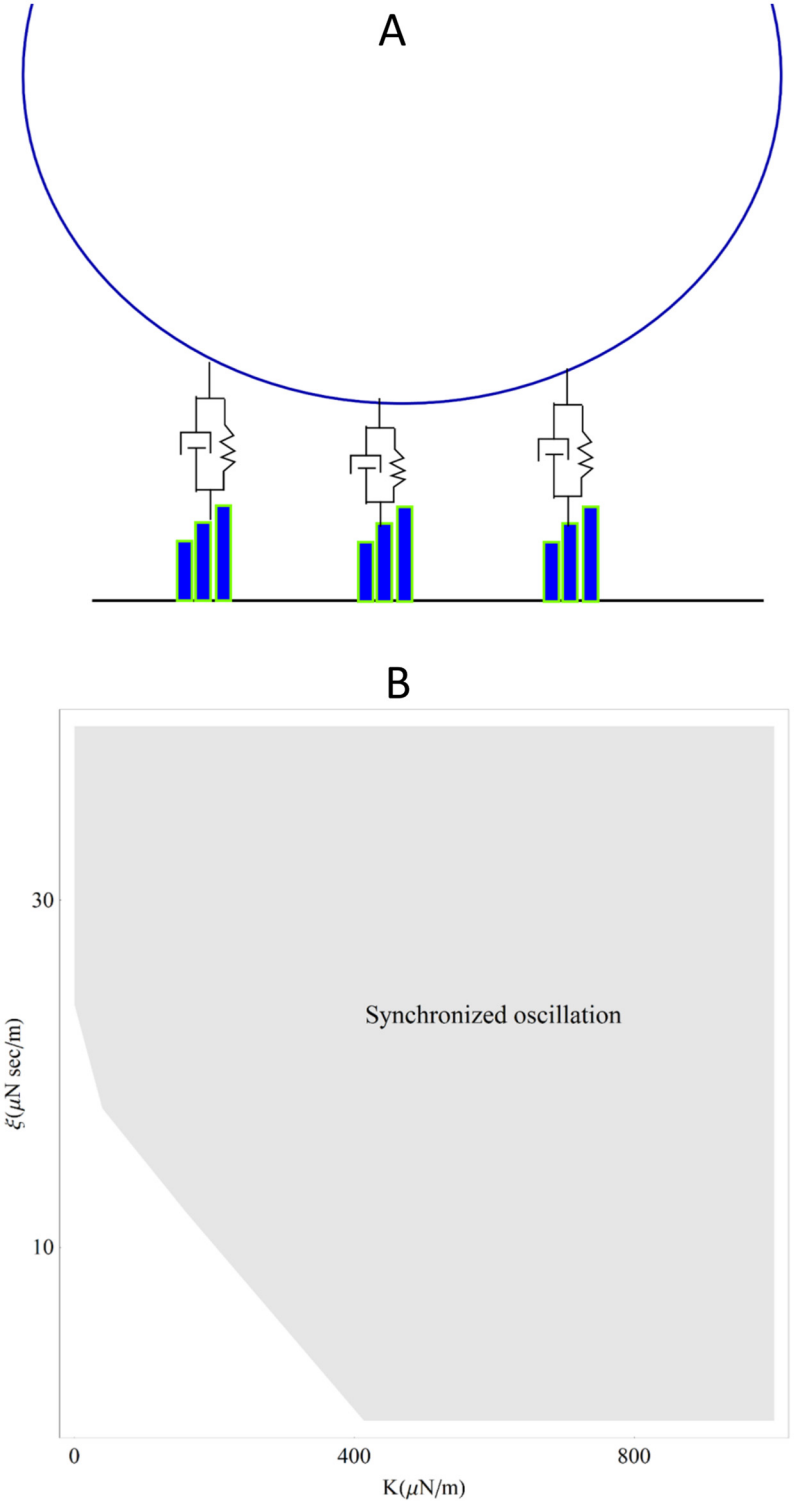
Synchronization in the theoretical model. **(A)** In this schematic, three non-linear oscillators are coupled by a bead via elastic and/or viscous coupling. **(B)** The plot shows the synchronization regime when μ = 1000 μN/m and λ = 2.8 μN sec/m. Synchronization is defined by the condition: Max[(Ω_1_, Ω_2_, Ω_3_, Ω_bead_)]-Min[(Ω_1_, Ω_2_, Ω_3_, Ω_bead_)]< 0.05 Max[(Ω_1_, Ω_2_, Ω_3_, Ω_bead_)]. In the region shown in the plot, frequencies are calculated for 2000 points in the phase space, to determine the synchronization boundary. Roughly, K>500 μN/m or ξ > 20 μN*s/m are required to synchronize the oscillators.

The coupled system is modeled with three nonlinear oscillators, connected to an overlying sphere (see schematic in Fig 5A). We introduce dimensions into our model:

$$\left(\lambda +\xi \right)\frac{\text{d}x_{i}}{\text{dt}}=\mu_{i}x_{i}-\omega_{i}y_{i}-f\left(x_{i}{}^{2}+y_{i}{}^{2}\right)x_{i}-K\left(x_{i}-M\right)+\xi \frac{\text{dX}}{\text{dt}}$$

$$\lambda \frac{\text{d}y_{i}}{\text{dt}}=\mu_{i}y_{i}+\omega_{i}x_{i}-f\left(x_{i}{}^{2}+y_{i}{}^{2}\right)y_{i}$$

$$M\frac{d^{2}X}{{\text{dt}}^{2}}=-\left(\Gamma +3\xi \right)\frac{\text{dX}}{\text{dt}}-3KX+K\left(x_{1}+x_{2}+x_{3}\right)+\xi \left(\frac{\text{d}x_{1}}{\text{dt}}+\frac{\text{d}x_{2}}{\text{dt}}+\frac{\text{d}x_{3}}{\text{dt}}\right)$$

where μ and K have the units of a spring constant (N/m), ξ and λ have the units of a friction coefficient (μN*s/m), and *f* has the units of force density(N/m^3^). The spontaneous oscillation frequency is Ω = ω/λ, and the spontaneous oscillation amplitude is $\sqrt{\frac{\mu }{f}}$. The chosen values of the parameters are based on the physiological characteristics of a hair cell (see Table A in S1 File).

We assume that the internal dynamics of a hair cell are not directly coupled to the bead; we hence introduce elastic and viscous coupling only between the sphere (*X)* and the real components of the motion of the oscillators (*x*_*i*_).

### Synchronization

Non-linear oscillators can be synchronized by either elastic or viscous coupling. In Fig 5B, we define the oscillations to be synchronized, if all four peak frequencies (three oscillators and the bead) are within a 5% range. The result shows the K and ξ values required to yield synchronization of the bundles. Synchronization occurs when the elastic coupling strength is comparable to or higher than the bundle stiffness, or when the viscous coupling constant is comparable to or higher than the friction coefficient of the bundle.

**Fig 6:**
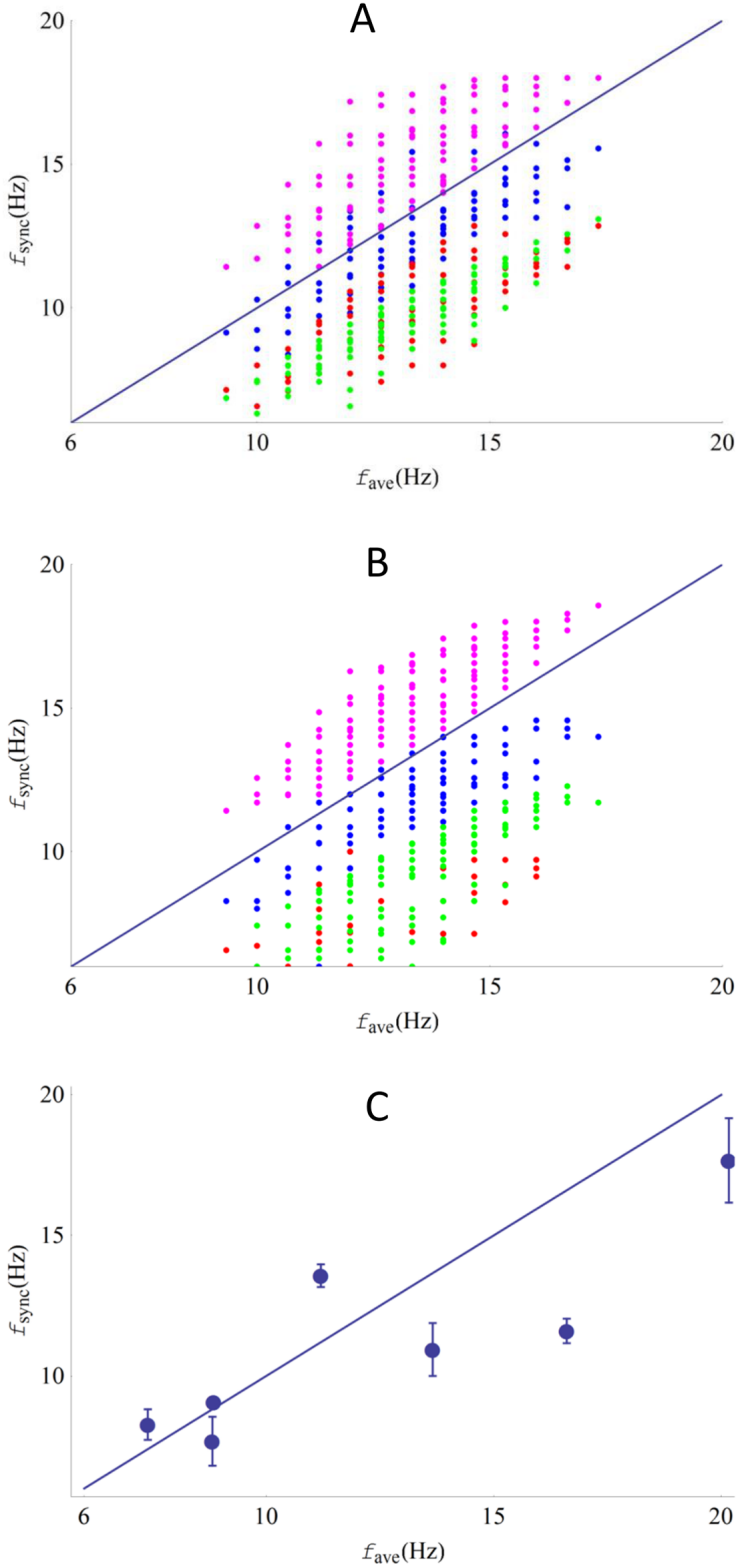
Synchronized frequency versus the average frequency of the bundles. **(A-B)** Numerical calculations of the synchronized frequency (*f*_*sync*_) of three bundles versus the average of their characteristic frequencies (*f*_*ave*_). Each data point represents a group of bundles, with a specific distribution of frequencies. The color coding represents different sets of parameter values, which include negative stiffness (μ) of the bundle, friction coefficient (λ), and coupling strength, while all other parameters in the model are fixed (see Table A in S1 File). **(A)** Elastic coupling (Blue: λ = 2.8 μN*s/m, K = 1000 μN/m, Red: λ = 0.28 μN*s/m, K = 100 μN/m, Green: λ = 0.28 μN*s/m, K = 1000 μN/m, Magenta: λ = 28 μN*s/m, K = 10000μN/m). **(B)** Viscous coupling (Blue: λ = 2.8 μN*s/m, ξ = 40 μN*s/m, Red: λ = 0.28 μN*s/m, ξ = 4 μN*s/m, Green: λ = 0.28 μN*s/m, ξ = 40 μN*s/m, Magenta: λ = 28 μN*s/m, ξ = 400μN*s/m). The simulations indicate that the synchronized frequencies are clustered near the average frequency values. However, precise values of the synchronized frequency depend on the characteristics of the bundles. **(C)** Experimental data. The error bars indicate the standard deviation for the three synchronized bundle frequencies.

Fig 6 plots the frequency of the synchronized system as a function of the mean frequency of the individual bundles. Results are presented for elastic (A) and viscous (B) coupling, with different distributions of frequencies of the individual oscillators, and different values chosen for the negative stiffness and friction coefficient of a bundle. Experimental results are shown in part (C). The theoretical results show clustering of the synchronized frequency to the mean frequency of the individual oscillators. The variation around this mean is due to its dependence on the stiffness and friction coefficients of the bundles.

### Phase lags

In our model, the hair bundles are assumed to be coupled only via the overlying spherical mass, rather than through any direct coupling among the bundles. As the spherical mass is not attached to any external structures, it moves in phase with the viscous force and exhibits a phase lag with respect to the elastic force. Hence, the two modes of coupling show different phase delays. A purely viscous force will lead to synchronization with zero phase differences among the bundles. On the other hand, purely elastic coupling can synchronize oscillations with a non-zero phase differences. S5 Fig panel (D) and (E) show the phase differences between the three oscillators as K or ξ is varied, demonstrating the difference between the two types of coupling. When the bundles are coupled by a weak elastic force, phase differences arise among the oscillators (S5A and S5D Fig), whereas phase differences are effectively zero when the oscillators are synchronized by even weak viscous coupling (S5 Fig panels (B) and (E)). However, the phase differences decrease to zero as the elastic coupling strength increases, and the response becomes indistinguishable from viscous coupling. Synchronization can be induced by various combinations of elastic and viscous coupling. S5 Fig panel (C) shows the phase differences when the system is coupled by both viscous and elastic elements. The plot clearly shows that the phase differences can be reduced by elastic or viscous couplings, but the viscous coupling is more effective.

### Multi-Mode Locking

Nonlinear systems can exhibit multi-mode phase-locking to an external signal, with the order of the mode dependent on the frequency of the imposed stimulus. A plot of winding number vs. detuning typically shows the “Devil's staircase” structure [45]. For a system of coupled oscillators, the synchronization mode will depend not only on the detuning parameter, but also on the strength of the coupling coefficients.

**Fig 7:**
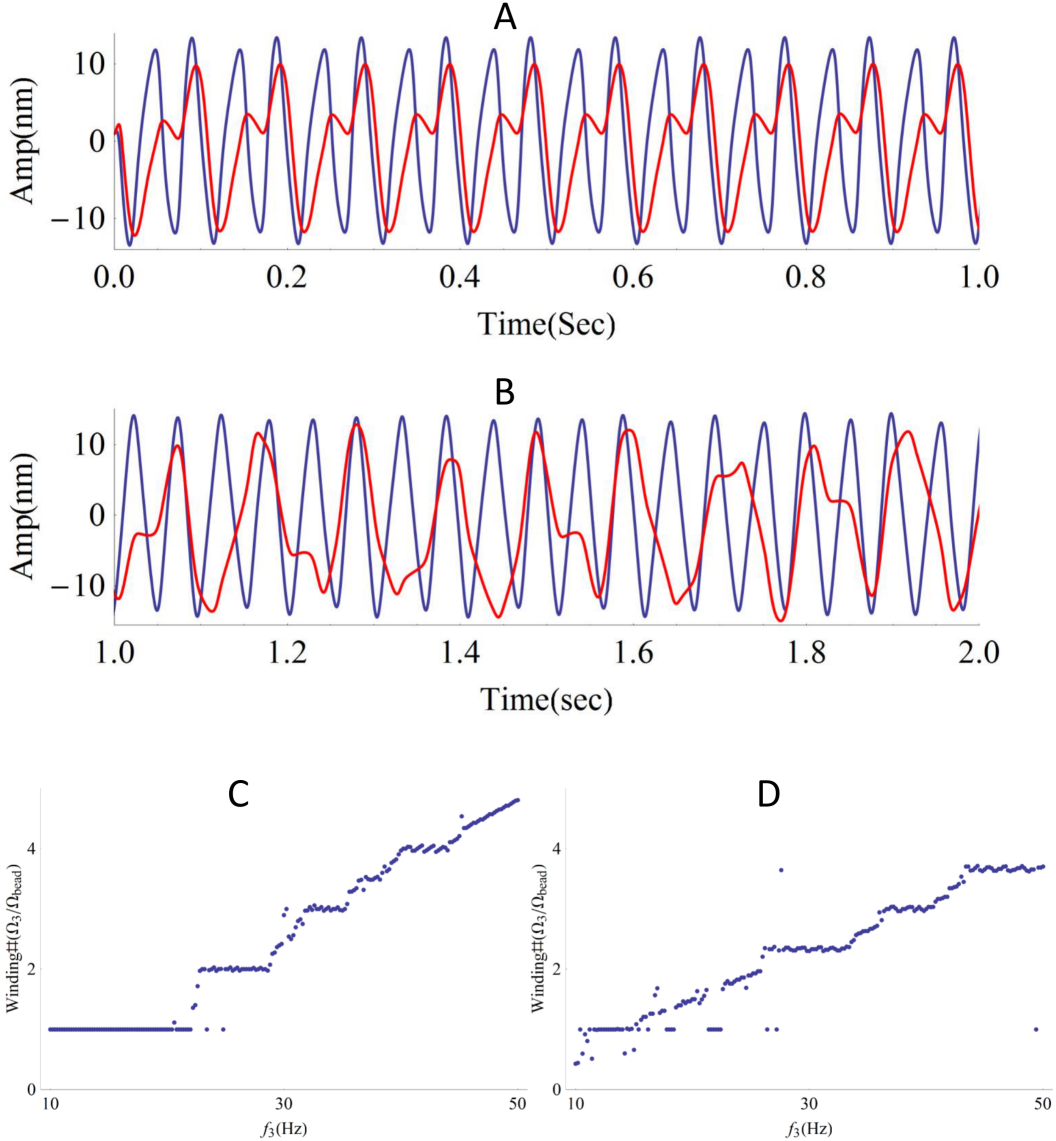
Multi-mode phase-locking by elastic or viscous coupling. **(A)** Multi-mode locking due to elastic coupling (Winding Number = 1.98). The red trace shows the bead motion, and the blue trace shows the motion of the hair bundle with the weaker coupling coefficient. The innate frequencies of the three oscillators are Ω_1_ = 7Hz, Ω_2_ = 17Hz and Ω_3_ = 25Hz; the coupling strength is K_1_ = 1000 μN /m, K_2_ = 1000 μN /m and K_3_ = 300 μN /m. **(B)** Multi-mode locking due to viscous coupling (Winding Number = 2.06). The red trace shows the bead motion, and the blue trace shows the motion of the hair bundle with the weaker coupling coefficient. The innate frequencies of the three oscillators are Ω_1_ = 7Hz, Ω_2_ = 17Hz and Ω_3_ = 25 Hz; the coupling strength is ξ_1_ = 40 μN*s/m, ξ_2_ = 40 μN*s/m and ξ_3_ = 2 μN*s/m. Both forms of coupling lead to multi-mode phase-locking. **(C-D)** Winding Number vs. frequency of one of the oscillators. Both forms of coupling show the devil’s staircase. **(C)** K_1_ = K_2_ = 1000 μN /m and K_3_ = 300 μN /m, Ω_1_ = 7 Hz, Ω_3_ = 17 Hz. **(D)** ξ_1_ = ξ_2_ = 40 μN*s/m, and ξ_3_ = 5 μN *s/m, Ω_1_ = 7 Hz, Ω_3_ = 17 Hz.

In Fig 7A and 7B, we plot traces of motion for one of the three coupled oscillators and the spherical mass, with the coupling strength of one of the oscillators assumed to be weaker than the other two. Both purely elastic and viscous coupling produce clear multi-mode phase-locking. Variations in the frequency of the oscillator with the weaker coupling coefficient lead to the devil's staircase (Fig 7C and 7D). The experimentally observed multi-mode locking is readily reproduced by the numerical simulation, indicating that the nonlinearity of the system is well described by the model.

## Discussion

### Synchronization of innate motility

The auditory system detects mechanical signals as weak as 0 dB SPL. The system is also robust, with the dynamic range of detectable sound pressures spanning over 6 orders of magnitude [5, 46]. It has been shown that the sensitivity and robustness of the inner ear require a nonlinear response. Many studies further indicate the presence of an underlying active mechanism that amplifies the mechanical response. SOAEs *in vivo* and active hair bundle oscillations *in vitro* are two of the signatures indicating the presence of an energy-consuming process. Connections between the two phenomena have however not yet been established.

Our results demonstrate synchronization between spontaneously oscillating hair cell bundles of the inner ear. These experiments confirm theoretical predictions for synchronization under coupled conditions [33], in a biological preparation that maintains the functional integrity of the hair cells. Coherent active motility of the bundles was clearly sufficient to drive the oscillations of the overlying bead in a viscous fluid environment. Synchronization was observed in the systems studied, despite significant dispersion of the characteristic frequencies of the constituent oscillators.

### Coupling elements in auditory and vestibular systems

Saccular hair bundles *in vivo* are coupled by the overlying otolithic membrane, a 25–30 mm thick matrix of densely packed randomly cross-linked filaments [47]. Hair bundles were shown to constitute the dominant elastic component of the lateral shearing of the otolithic membrane [40, 48]. Consistent with these findings, we observed in a prior study that a localized mechanical perturbation elicited a coherent response across hundreds of cells [40]. Comparable results were obtained in other species: a patch of tectorial membrane isolated from the mouse cochlea was ~10 times stiffer than the aggregate of the spanned bundles [49]. In the current study, the compliance of the polystyrene sphere was negligible with respect to the bundles, approximating the properties of coupling structures *in vivo*.

Comparatively little is known about the viscoelastic properties of the connections between the bundles and the overlying membranes. In the sacculus, thin linkages were observed to connect the kinocilia to pits in the otolithic membrane [47]. A comparative study of these connecting elements across the species, and their effects on synchronization, has not been performed.

Our artificial coupling element allowed us to experimentally study a specific range of coupling coefficients, weaker than those observed in the semi-intact saccular preparation [50]. The numerical model indicated that, for small groups of coupled hair cells, synchronization should arise over a broad range of coupling coefficients. This implies that synchronized active motility could be observed in a number of different species.

### Frequency clustering

Frequency clustering of coupled oscillators was explored in a theoretical study of spontaneous otoacoustic emissions [34]. Different modes of coupling were shown to lead to different patterns in the observed frequencies of oscillation of the synchronized clusters. With connections between the individual elements primarily elastic, frequency of the entrained group of oscillators coincided with the highest frequency within the cluster. In contrast, with dissipative coupling between the oscillators, entrained motion was shown to occur at the mean frequency of the cells.

To estimate the effects of coupling coefficients on the synchronized frequency, we modeled our system with three oscillatory hair bundles, exhibiting different innate frequencies, coupled by an overlying sphere. We found that either elastic or viscous coupling could lead to synchronization frequencies close to the mean of the individual oscillators. The lack of a significant phase lag indicated a significant viscous component in the coupling coefficient.

### Quality factors

Hair bundles oscillated more regularly when coupled to the overlying bead. The quality factors of the coupled bundles were ~1.2–1.8x larger than those of the individual bundles. Slightly higher enhancement (2x) was observed in the system where one hair bundle was coupled to its cyber clone [36]. The theoretical model for a coupled system of 2x2 identical oscillators showed a 4-5x increase in the quality factor under conditions of strong coupling [33]. The enhancement was therefore higher than what was observed in our hybrid preparations. We ascribe the discrepancy to the frequency dispersion of the biological system. In both of the prior studies, the coupled bundles were chosen to exhibit identical innate frequencies, whereas the saccular epithelium displays broad frequency dispersion [40]. The enhancement of the quality factor, present despite dispersion in the frequencies of constituent oscillators, indicates the importance of coupling in shaping the frequency tuning of the whole system.

In addition to synchronizing the spontaneous oscillations, the overlying microsphere also imposed a mass load on the hair bundles. The 50 mm polystyrene sphere yields ~60 ng of mass; for comparison, the mass of a hair bundle is estimated at ~60 pg [21]. The effects of mass on the nonlinear response has not been extensively addressed in theoretical studies of the coupled system, but is likely to play a role in determining the overall quality factor of naturally coupled hair bundles *in vivo*.

### Potential implications for *in vivo* phenomena

Spontaneous oscillation [21] exhibited by a hair cell’s stereovillar bundle constitutes a potential cellular mechanism underlying the phenomenon of spontaneous otoacoustic emission [30–32]. However, these active oscillations have been mostly studied in the uncoupled bundles of the amphibian sacculus. The presence of the overlying otolithic membrane, which strongly loads and couples the bundles across the full epithelium, was shown to inhibit innate oscillations [38]. The question of whether different coupling conditions could lead to synchronized active bundle motility, thus providing a potential mechanism for *in vivo* emissions, has remained open. The use of a hybrid preparation, in which a small number of hair cells are artificially connected, provides us with a model system, wherein we can mimic coupling in other species. In particular, many species of lizards have been shown to have robust emissions [31]; in a number of lizard papillae, small numbers of hair cells are connected to patches of membrane known as sallets. Coupling between hair bundles is ubiquitous in inner ear end organs of many other species as well, including mammalian cochleae, where an overlying tectorial membrane couples hair bundles over various spatial scales [32]. Our findings demonstrate that synchronized active motility of a small number of hair cell bundles could power oscillations in significantly larger overlying structures. Spontaneous bundle motility hence constitutes a plausible mechanism for the generation of sound, which could be emitted via the reverse auditory pathway.

## Supporting Information

S1 Fig Quality factors of the oscillation traces. **(A)** A typical recording of the innate motility exhibited by an oscillating hair bundle. The superposed square wave trace represents the rapid positive and negative deflections of the bundle, obtained from the oscillation detection program, described in Methods. The interval between two positive deflections defines the instantaneous period of the cycle. Scale bar x = 200 ms, y = 20 nm. **(B)** Kernel density estimation (KDE) of instantaneous frequencies, obtained from the inverse of the instantaneous period. The position of the peak of the curve defines the frequency F_peak_, of the bundle, and the Q-factor is defined as F_peak_/FWHM. **(C)** KDE of instantaneous frequencies, with the instantaneous period defined by the interval between two negative deflections. No significant differences were observed between the two methods.
(TIFF)

S2 Fig Characteristics of the correlation coefficient. **(A)** Distribution of the bundle-bead correlation coefficients obtained from eight recordings. All bundles within 30 μm of the bead center, including those immediately outside the rim (at 25 μm), are included. The distribution shows clustering into more strongly and weakly correlated bundles. The color code categorizes bundles into synchronized (1:1 mode-locked), multi-mode locked, and not synchronized. We chose 0.5 as the cutoff for synchronization. **(B)** Fluctuation of the bundle-bead correlation coefficient over time, for a typical bundle. The recording was 11 seconds long and was divided into time windows of 0.5 seconds each. The data points in the plot represent the bundle-bead correlation in each time window. x scale bar = 1 second.
(TIFF)

S3 Fig Distribution of bundle positions. The plot collects xy-positions of hair bundles, obtained from eight recordings. The orange dashed line represents the 50μm bead, with the bead center at the center of the crosshair. The bundles are categorized as synchronized (1–1 mode-locked), multimode-locked, and not synchronized. The majority of the bundles within 16μm from the bead center were synchronized, and the majority of all synchronized bundles were within this range.
(TIFF)

S4 Fig Method of measuring k and ξ. **(A)** A schematic diagram of the coupling between the bead, the bundle, and the supporting tissue. **(B)** A typical distribution of k(⍵) for a single bundle-bead pair. The kernel density estimation curve (KDE) provides a peak value, and the width at half maximum gives the error estimate +/-Δk.
(TIFF)

S5 Fig Phase lags and the coupling coefficients in the numerical simulation (Logarithmic scale). **(A)** The calculated traces of motion for hair bundles coupled by an elastic element, with coupling strength K = 500μN/m. The traces represent the motion of the bundles and the bead (Motion of the bundles: Red, Green Magenta, Motion of bead: Blue). Other parameter values are shown in Table A in S1 File. The oscillators are synchronized with non-zero phase differences. Δφ = *φ*_bead_ − *φ*_*i*_. **(B)** The calculated traces of motion for hair bundles coupled by a viscous element, with coupling strength ξ = 50μN*s/m. The traces represent the motion of the bundles and the bead (Motion of the bundles: Red, Green Magenta, Motion of the bead: Blue). The oscillators are in phase. **(C)** Phase difference (Δφ = *φ*_bead_ − *φ*) map of a hair bundle, as a function of ξ and K (Ω_1_ = 7 Hz, Ω_2_ = 17Hz, Ω_3_ = 25Hz; μ = 1000 μN/m and λ = 2.8μN sec/m). The phase lag is obtained from the peaks in the Fourier transforms of the oscillation traces. The phase delay is reduced to zero either by viscous coupling or by strong elastic coupling (K> 10^-2^N/m). **(D-E)** The three curves in each plot show the phase lags between the bead and the three oscillators. Phase values are calculated from the Fourier transforms at the synchronized frequency. The two plots show the differences between elastic and viscous couplings. **(D)** Purely elastic coupling (ξ = 0). **(E)** Purely viscous coupling (K = 0). **(F)** Lower bound of the viscous coupling strength versus elastic coupling strength. K and ξ values are normalized by μ and λ. The parameter values are chosen to be: Ω_1_ = 7 Hz, Ω_2_ = 17Hz, Ω_3_ = 23 Hz; μ = 100, 1000 or 10000 μN/m and λ = 0.28, 2.8 or 280 μN*s/m. All combinations of the parameter values are investigated (9 combinations of parameters: μ = 10000, λ = 0.28(●),μ = 10000, λ = 2.8 (Δ),μ = 1000, λ = 0.28(♦)μ = 1000, λ = 2.8 (□),μ = 100, λ = 0.28(○),μ = 100, λ = 2.8 (▲),μ = 10000, λ = 28(▼),μ = 1000, λ = 28(◊),μ = 100, λ = 28(■)) for the K values within the range of 0 < K < 10000 μN/m (17 K values). Each point is the lower bound for ξ that leads to phase lag less than 0.2 radians.
(TIFF)

S1 File Supplement. (DOCX)
