# Supplementary figures and images for: Synchronization of Spontaneous Active Motility of Hair Cell Bundles
Source: PLoS One. 2015 Nov 5;10(11):e0141764. doi: 10.1371/journal.pone.0141764 (PMC4634766; doi:10.1371/journal.pone.0141764)

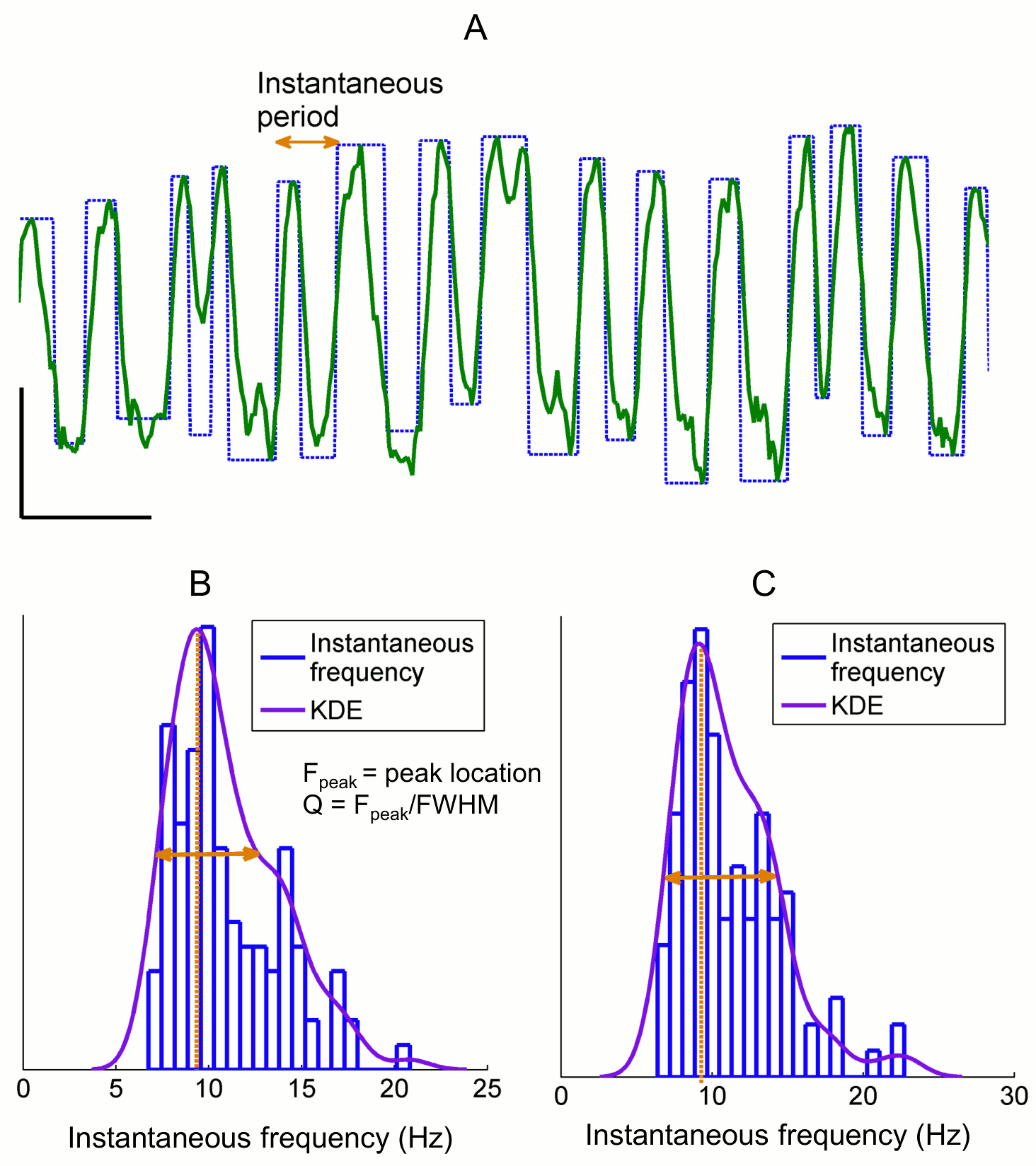

Supplement: S1 Fig — (A) A typical recording of the innate motility exhibited by an oscillating hair bundle. The superposed square wave trace represents the rapid positive and negative deflections of the bundle, obtained from the oscillation detection program, described in Methods. The interval between two positive deflections defines the instantaneous period of the cycle. Scale bar x = 200 ms, y = 20 nm. (B) Kernel density estimation (KDE) of instantaneous frequencies, obtained from the inverse of the instantaneous period. The position of the peak of the curve defines the frequency Fpeak, of the bundle, and the Q-factor is defined as Fpeak/FWHM. (C) KDE of instantaneous frequencies, with the instantaneous period defined by the interval between two negative deflections. No significant differences were observed between the two methods. (TIFF) [file pone.0141764.s001.tiff]

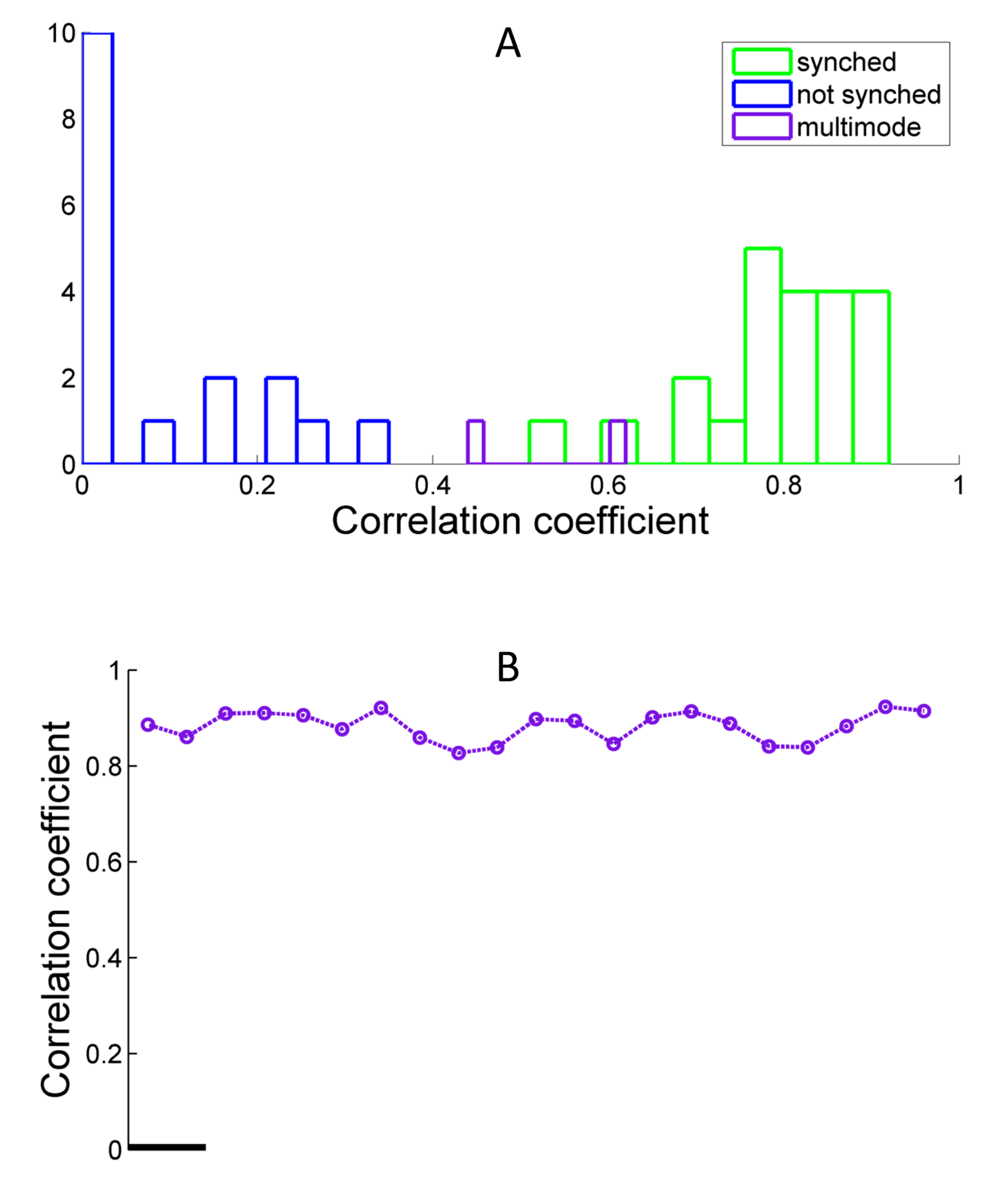

Supplement: S2 Fig — (A) Distribution of the bundle-bead correlation coefficients obtained from eight recordings. All bundles within 30 μm of the bead center, including those immediately outside the rim (at 25 μm), are included. The distribution shows clustering into more strongly and weakly correlated bundles. The color code categorizes bundles into synchronized (1:1 mode-locked), multi-mode locked, and not synchronized. We chose 0.5 as the cutoff for synchronization. (B) Fluctuation of the bundle-bead correlation coefficient over time, for a typical bundle. The recording was 11 seconds long and was divided into time windows of 0.5 seconds each. The data points in the plot represent the bundle-bead correlation in each time window. x scale bar = 1 second. (TIFF) [file pone.0141764.s002.tiff]

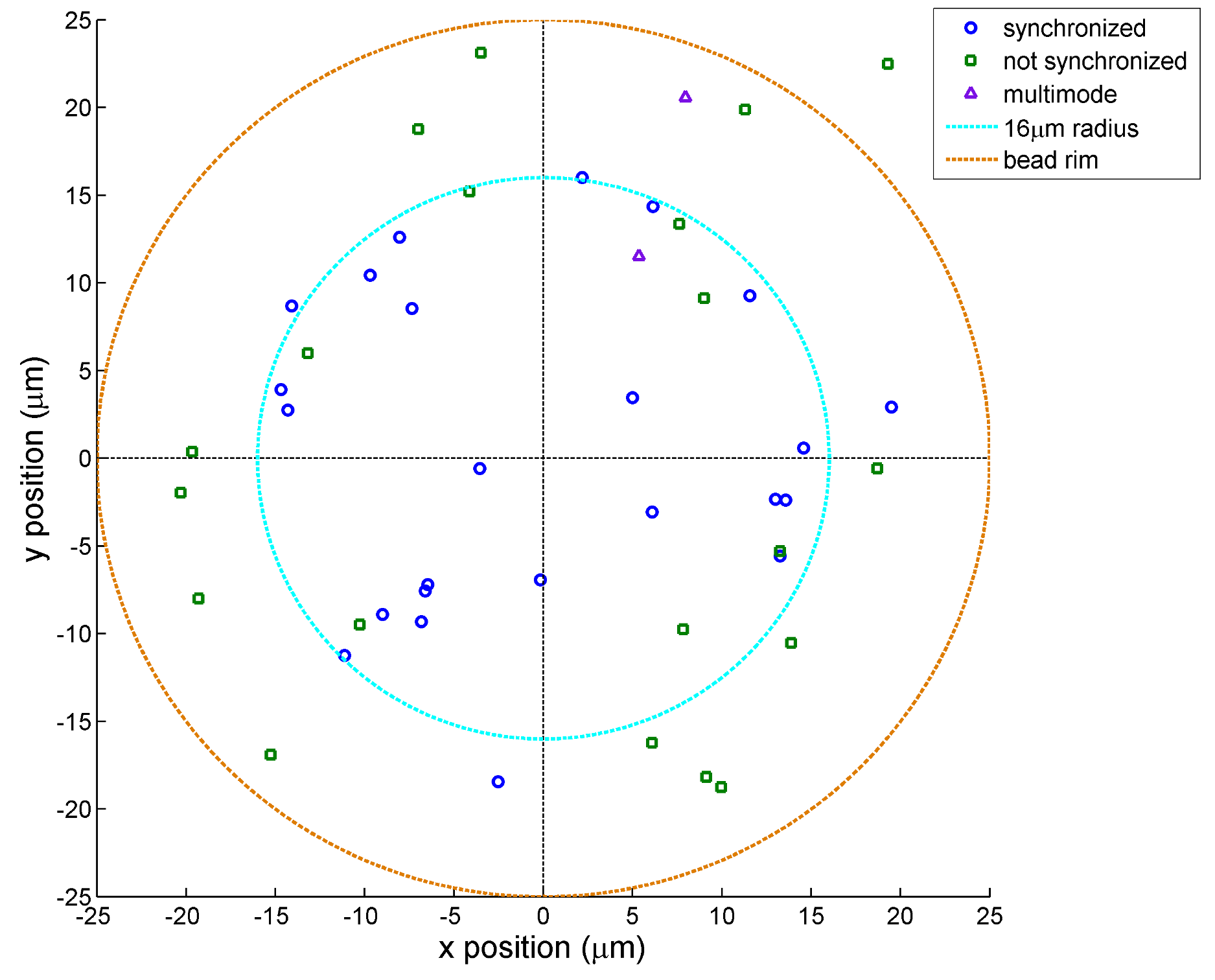

Supplement: S3 Fig — The plot collects xy-positions of hair bundles, obtained from eight recordings. The orange dashed line represents the 50μm bead, with the bead center at the center of the crosshair. The bundles are categorized as synchronized (1–1 mode-locked), multimode-locked, and not synchronized. The majority of the bundles within 16μm from the bead center were synchronized, and the majority of all synchronized bundles were within this range. (TIFF) [file pone.0141764.s003.tiff]

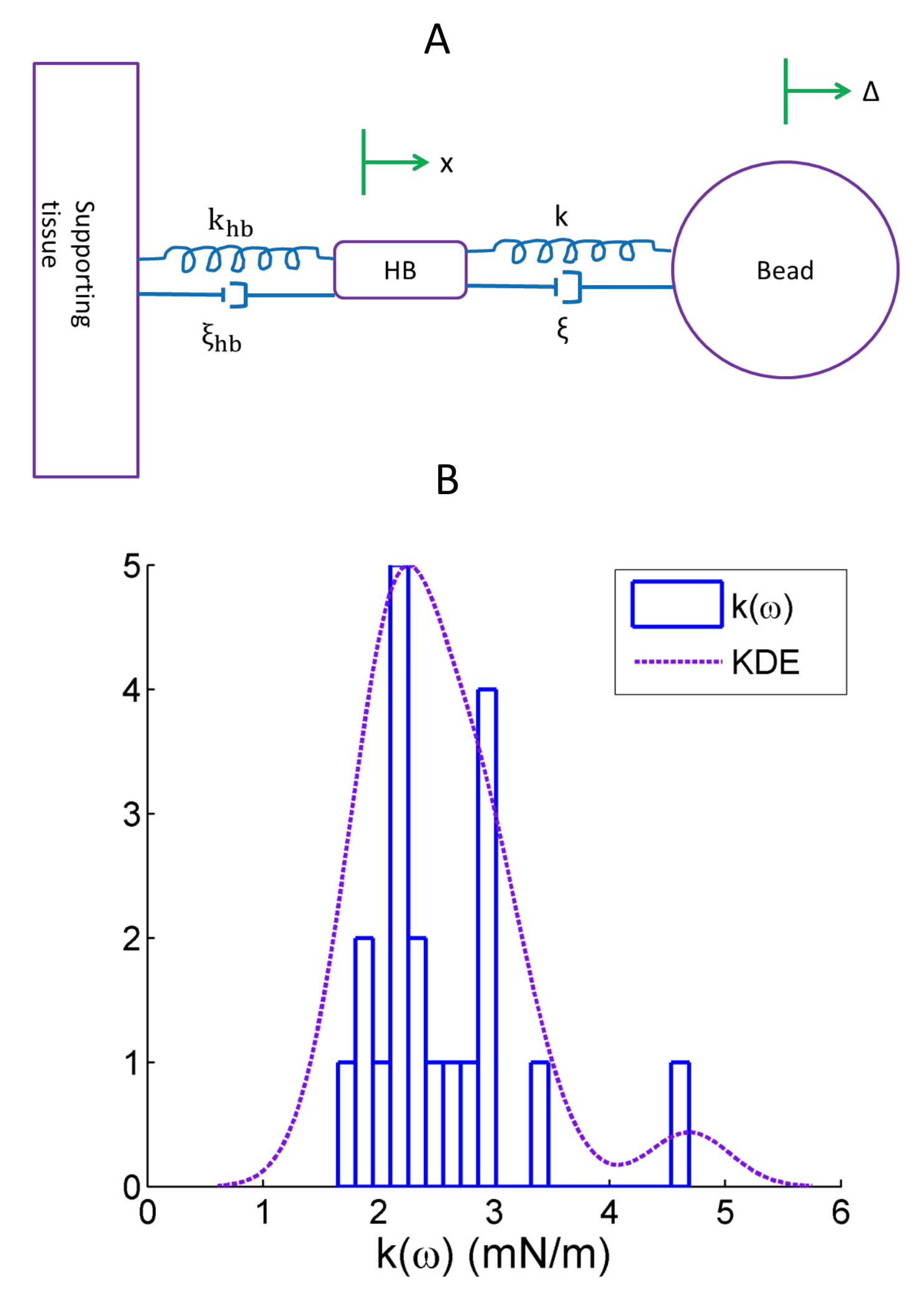

Supplement: S4 Fig — (A) A schematic diagram of the coupling between the bead, the bundle, and the supporting tissue. (B) A typical distribution of k(⍵) for a single bundle-bead pair. The kernel density estimation curve (KDE) provides a peak value, and the width at half maximum gives the error estimate +/-Δk. (TIFF) [file pone.0141764.s004.tiff]

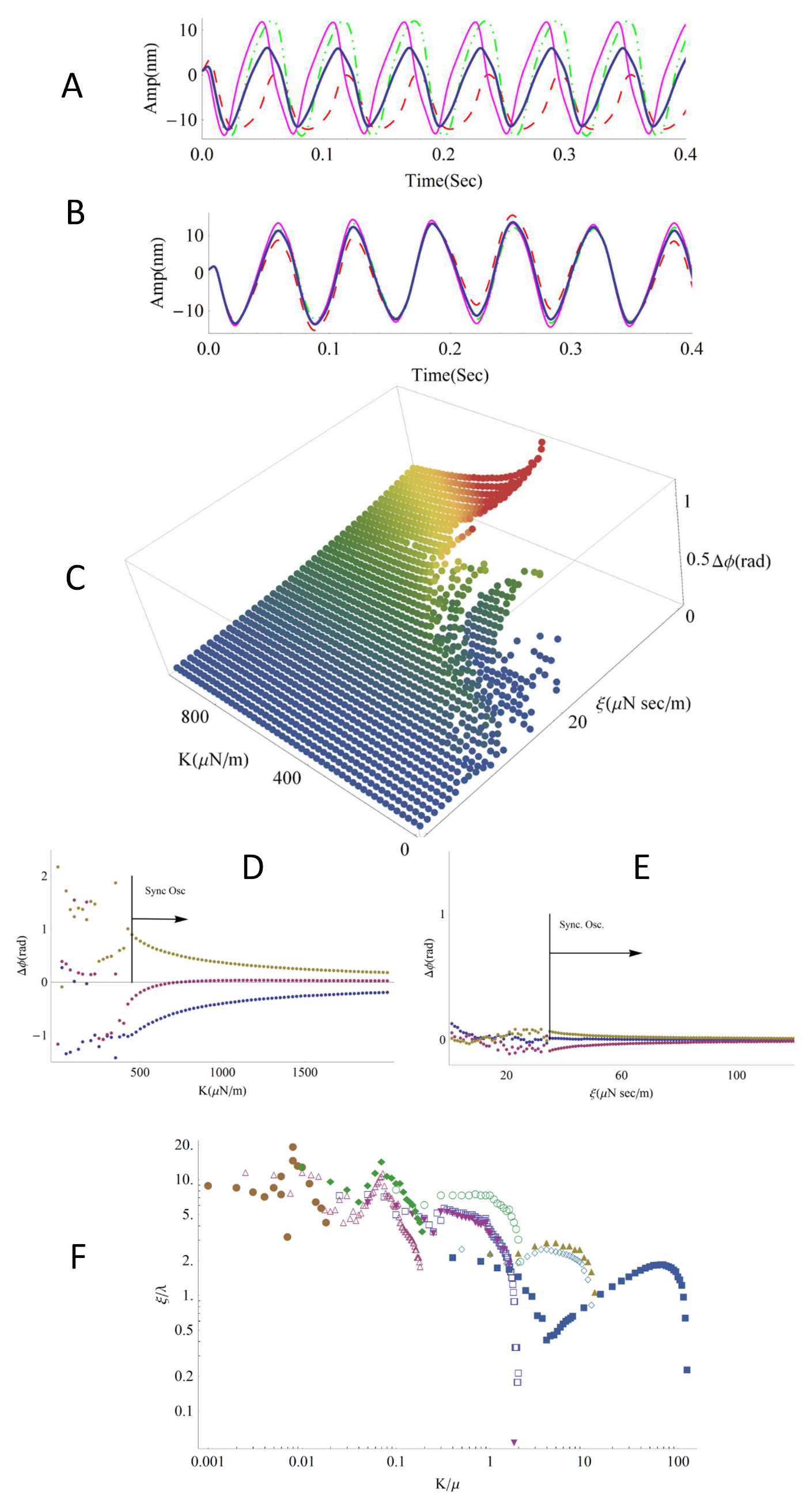

Supplement: S5 Fig — (A) The calculated traces of motion for hair bundles coupled by an elastic element, with coupling strength K = 500μN/m. The traces represent the motion of the bundles and the bead (Motion of the bundles: Red, Green Magenta, Motion of bead: Blue). Other parameter values are shown in Table A in S1 File. The oscillators are synchronized with non-zero phase differences. Δφ = φ bead − φ i. (B) The calculated traces of motion for hair bundles coupled by a viscous element, with coupling strength ξ = 50μN*s/m. The traces represent the motion of the bundles and the bead (Motion of the bundles: Red, Green Magenta, Motion of the bead: Blue). The oscillators are in phase. (C) Phase difference (Δφ = φ bead − φ) map of a hair bundle, as a function of ξ and K (Ω1 = 7 Hz, Ω2 = 17Hz, Ω3 = 25Hz; μ = 1000 μN/m and λ = 2.8μN sec/m). The phase lag is obtained from the peaks in the Fourier transforms of the oscillation traces. The phase delay is reduced to zero either by viscous coupling or by strong elastic coupling (K> 10-2N/m). (D-E) The three curves in each plot show the phase lags between the bead and the three oscillators. Phase values are calculated from the Fourier transforms at the synchronized frequency. The two plots show the differences between elastic and viscous couplings. (D) Purely elastic coupling (ξ = 0). (E) Purely viscous coupling (K = 0). (F) Lower bound of the viscous coupling strength versus elastic coupling strength. K and ξ values are normalized by μ and λ. The parameter values are chosen to be: Ω1 = 7 Hz, Ω2 = 17Hz, Ω3 = 23 Hz; μ = 100, 1000 or 10000 μN/m and λ = 0.28, 2.8 or 280 μN*s/m. All combinations of the parameter values are investigated (9 combinations of parameters: μ = 10000, λ = 0.28(●),μ = 10000, λ = 2.8 (Δ),μ = 1000, λ = 0.28(♦)μ = 1000, λ = 2.8 (□),μ = 100, λ = 0.28(○),μ = 100, λ = 2.8 (▲),μ = 10000, λ = 28(▼),μ = 1000, λ = 28(◊),μ = 100, λ = 28(■)) for the K values within the range of 0 < K < 10000 μN/m (17 K values). Each point is the lower bound [file pone.0141764.s005.tiff]
