# Supplementary material for: Synchronization of Spontaneous Active Motility of Hair Cell Bundles
Source: PLoS One. 2015 Nov 5;10(11):e0141764. doi: 10.1371/journal.pone.0141764 (PMC4634766; doi:10.1371/journal.pone.0141764)
Supplement: S1 File — (DOCX) [file pone.0141764.s006.docx]

**Supplement**

**Position distribution of synchronized hair bundles**

In images obtained at the focal planes of the stereovilli, both the hair bundles and the overlying beads were visible (Fig 1C). The synchronized bundles were within the rim of the bead. To quantify the pattern of synchronization, we plotted the positions of hair bundles from eight recorded groups (S3 Fig). Most of the synchronized bundles (83%) were within a 16 µm radius of the bead center. Several bundles deviated from this pattern, likely due to the inherent variation in the heights of the bundles.

**Estimates of the coupling coefficients k and ξ**

The coupling between the bead and an underlying bundle can be modeled with viscoelastic elements, as shown in the schematic (S4 Fig panel (A)).

The viscoelasticity of the hair bundle (hb) is characterized by a stiffness $k_{hb}$ and viscous drag $\xi_{hb}$. Similarly, the connection between the bead and a hair bundle (through concanavalin A) is characterized by k and ξ. With a stimulus imposed on the bead, Δ is the displacement of the bead, and x is the displacement of the bundle. If the stimulus is sinusoidal, we can let $\Delta=\Delta_{0}e^{i⍵t}$ and $x= x_{0}e^{i⍵t+\varphi}$ be the response of the bead and bundle, respectively.

The force on the hair bundle is

$$F=m_{hb}\ddot{x}= \xi_{hb}\dot{x}+ k_{hb}x+ \xi\left( \dot{x}- \dot{\Delta} \right)+k\left( x-\Delta\right) (1)$$

Since the mass of the hair bundle $m_{hb}$ is negligible, the equation of motion is

$\xi_{hb}\dot{x}+ k_{hb}x+ \xi\left( \dot{x}- \dot{\Delta} \right)+k\left( x-\Delta\right)=0 (2)$

With sinusoidal stimulus, this becomes

$\left\{ \begin{aligned} \left( k_{hb}+k \right)x_{0}\cos\varphi-\left( \xi_{hb}+ \xi\right)⍵x_{0}\sin\varphi=k\Delta_{0} \\ \left( k_{hb}+k \right)x_{0}\sin\varphi+\left( \xi_{hb}+ \xi\right)⍵x_{0}\cos\varphi=⍵\xi\Delta_{0} \end{aligned} \right. (3)$

With the substitution

$A_{1}= x_{0}\cos\varphi-\Delta_{0} A_{2}= -⍵x_{0}\sin\varphi$ $A_{3}= k_{hb}x_{0}\cos\varphi-⍵\xi_{hb}x_{0}\sin\varphi$

$B_{1}= x_{0}\sin\varphi B_{2}= {⍵x}_{0}\cos\varphi-⍵\Delta_{0} B_{3}= k_{hb}x_{0}\sin\varphi+⍵\xi_{hb}x_{0}\cos\varphi$

The solutions are

$\left\{ \begin{aligned} k= \frac{B_{3}A_{2}-A_{3}B_{2}}{A_{1}B_{2}- B_{1}A_{2}} \\ \xi= \frac{B_{3}A_{1}- A_{3}B_{1}}{A_{2}B_{1}- B_{2}A_{1}} \end{aligned} \right. (4)$

Therefore, with a sinusoidal stimulus, we can measure the quantities ⍵, $x_{0}$, $\Delta_{0}$, and $\varphi$, and calculate k and ξ.

For this measurement, we sent a stimulus to the bead with a glass capillary probe, which was attached to the equator of the bead. Artificial endolymph was replaced by perilymph, so that the bundles were quiescent. We recorded the motion of individual bundles at the stereovillar focal plane. Spots adjacent to the bundles were also tracked, yielding the motion of the bead.

For the k measurement, the stimulus consisted of a series of sine wave segments, with frequency ranging from 5Hz to 100Hz. For each sine wave segment, we extracted ⍵, $x_{0}$, $\Delta_{0}$, and $\varphi$, which yielded a stiffness value for that frequency. The whole frequency sweep yielded a distribution (KDE) of stiffness values (S4 Fig panel (B)). The peak value of the smoothed curve was taken to be k value for this bundle-bead pair, and the width at half maximum gave the error +/-Δk. The results for 8 pairs yielded values from 1.5 (+1, - 0.7) to 4.8 (+/- 1.8) mN/m.

The same procedure was used to measure ξ, in the frequency range 550-650Hz. The results for 4 pair yielded values from 1.9 (+0.16, -0.1) to 3.2 (+/- 1.2) N*s/m.

The average values for the coupling coefficients are k = 2.5+/-1.1mN/m, and ξ = 2.8+/-0.7µN*s/m.

**Experimental constraints on the coupling coefficients in the numerical model**

Our experiments showed that the phase lags of the bundle motion with respect to that of the microsphere were within the time resolution of the recording (Δφ < 0.2 radian), for all synchronized hair cells. To obtain a lower bound for the viscous coupling coefficient, we performed simulations with various combinations of parameters, within physiological limits. The bundle’s negative stiffness (μ) and the friction coefficient (λ) were varied. In S5 Fig panel (F), we plot the minimum values of ξ (ξ_min_(K)), obtained for varying values of K, which satisfy the experimental limits on the phase lag. The result shows that the viscous coupling needs to be ~10 times higher than the friction coefficient of a hair bundle (ξ_min_>10 λ), if 0 < K < μ. When μ < K <10 μ, the lower bound for ξ is ~5 λ. When K is greater than ~10 times the negative stiffness of a bundle, viscous coupling is not necessary to obtain a zero phase lag, except in the extreme cases, with low negative stiffness and high bundle friction coefficient (K > 100 μ in order to obtain a zero phase lag without ξ , when μ~100 μN/m, λ~10 μN*s/m). Thus, if the elastic coupling is comparable to the negative stiffness of the bundle, viscous coupling is required to reach zero phase lag based on the theoretical model.

**Simulation Parameters**

Table A: Parameter values used in the simulation.

| Friction coefficient of a bundle | λ= 2.8 (Figs 5-7) , and 0.28, 2.8 and 28(Fig 6 & S5(F)) μN*s/m | Ref. 21 & 17 |
| --- | --- | --- |
| Negative Stiffness of a bundle | μ= 1000(Figs 6-7, S5) and 100, 1000,10000 (Fig S5(F)) μN/m | Ref. 21 |
| Radius of a bead | a = 25 μm |  |
| Mass of a bead | M = $5.5 \times{10}^{-7}$ g |  |
| Friction coefficient of a bead | Γ = 0.4722 μN*s/m |  |
| Non-linear coefficient | *f* = $5\times{10}^{12}$N/m^3^ | Calculated from dimensions |
| Elastic coupling strength | K=500, 1000 μN/m(Fig 6(A), S5(C)), 0 – 10000 μN/m(Fig S5(F)) and K=300, 1000 μN/m(Fig 7) |  |
| Viscous coupling strength | ξ = 4,40,400 μN*s/m(Fig 6(B)) , ξ = 50μN*s/m(Fig S5(B)),0-100 μN*s/m(Fig S5(F)), 2, 40 μN*s/m(Fig 7(B)) and 5, 40 μN*s/mFig (7(D)). |  |
